# Multiple Ventricular Septal Defects: A New Strategy

**DOI:** 10.3389/fped.2013.00016

**Published:** 2013-07-31

**Authors:** Antonio F. Corno, Pramod R. Kandakure, Ramana Rao V. Dhannapuneni, Gordon Gladman, Prem Venugopal, Nelson Alphonso

**Affiliations:** ^1^King Fahad Medical CityRiyadh, Saudi Arabia; ^2^Alder Hey Children’s NHS Foundation TrustLiverpool, UK

**Keywords:** congenital heart defects, multiple ventricular septal defects, palliation, pulmonary artery banding, surgical repair

## Abstract

**Introduction:** A multicenter prospective study was conducted to evaluate a new strategy for multiple Ventricular Septal Defects (VSDs).

**Materials and Methods:** From 2004 to 2012 17 consecutive children (3 premature, 14 infants), mean age 3.2 months (9 days–9 months), mean body weight 4.2 kg (3.1–6.1 kg), with multiple VSDs underwent Pulmonary Artery Banding (PAB) with an adjustable FloWatch-PAB^®^. Associated cardiac anomalies included patent ductus arteriosus (1), aortic coarctation (2), hypoplastic aortic arch (2), and left isomerism (3). Five patients (5/17 = 29.4%) required pre-operative mechanical ventilation, with a mean duration of 64 days (7–240 days)

**Results:** There were no early or late deaths during a mean follow-up of 48 months (7–98 months), with either FloWatch removal or last observation as end-points. FloWatch-PAB^®^ adjustments were required in all patients: a mean of 4.8 times/patient (2–9) to tighten the PAB, and a mean of 1.1 times/patient (0–3) to release the PAB with the patient’s growth. After a mean interval of 29 months (8–69 months) 10/17 (59%) patients underwent re-operation: 7/10 PAB removal, with closure of a remaining unrestrictive VSD in 6 (peri-membranous in 3 patients, mid-muscular in 2, and inlet in 1) and Damus–Kaye–Stansel, bi-directional Glenn, and atrial septectomy in 1; 3/9 patients required only PAB removal. All muscular multiple VSDs had closed in all 10 patients. PA reconstruction was required in 1/10 patient. In 5/7 of the remaining patients with the PAB still *in situ*, all muscular VSDs had already closed. The only 2 patients with persistent muscular multiple VSDs are the 2 patients with the shortest follow-up.

**Conclusion:** This reproducible new strategy with an adjustable PAB simplifies the management of infants with multiple VSDs and provides the following advantages: (a) good results (0% mortality), delayed surgery with a high incidence (15/17 = 88%) of spontaneous closure of multiple muscular VSDs, and facilitated closure of residual unrestrictive VSD (peri-membranous, mid-muscular, or inlet) at an older age and higher body weight; PAB with FloWatch-PAB^®^ and its subsequent removal can potentially be the only procedure required for Swiss cheese multiple VSDs without an associated peri-membranous unrestrictive VSD.

## Introduction

Multiple muscular Ventricular Septal Defects (VSDs) are defined as the presence of more than one VSD in the muscular septum, with or without other VSD located in the peri-membranous or outlet septum, with or without associated intra-cardiac or extra-cardiac malformations (3).

Conventional surgical repair of multiple muscular VSDs is generally accompanied by substantial early and late mortality and morbidity (1, 2, 4–21).

The hospital mortality with conventional repair has been reported in the literature to be between 0 and 14.2% (1, 2, 4–21). In the 192 patients reported in the Congenital Database of the European Association of Cardio-Thoracic Surgery the hospital mortality was 8.5%^1^.

In the previous decade an alternative approach with intra-operative device closure reported unsatisfactory initial overall results due to elevated mortality (14–25%) and high failure rates (20–40%) (22–25). More recently institutions with advanced facilities for hybrid techniques have reported encouraging outcomes in selected series of multiple VDSs closure (26–30).

A palliative approach with conventional pulmonary artery banding (PAB) is a common surgical option, particularly in smaller infants and/or in the presence of associated anomalies, instead of primary repair (1, 2, 4–10, 15, 16, 18–21, 31). However the conventional PAB carries a high mortality. The hospital mortality in the 739 patients reported in the Congenital Database of the European Association of Cardio-Thoracic Surgery (see text footnote 1) was 8.9%. In addition, indication for conventional PAB is limited by a series of drawbacks, particularly due to the difficulty in determining the optimal tightening with a stable and satisfactory balance between systemic and pulmonary circulation to promote adequate growth (1, 2, 4–10, 15, 16, 18–21, 30–37).

After prolonged experimental studies (32) we introduced the telemetrically adjustable FloWatch-PAB^®^ (Leman Medical Technologies, Lausanne, Switzerland) in the clinical practice in 2003. We and other institutions have since reported favorable clinical results with its utilization in various congenital heart defects (33–40).

This multicenter prospective study was conducted to evaluate a new strategy to treat infants with multiple VSDs with FloWatch-PAB^®^.

## Materials and Methods

From 2004 to 2012 17 consecutive children (3 premature, 14 infants), mean age 3.2 months (range 9 days–9 months), mean body weight 4.2 kg (range 3.1–6.1 kg), with multiple VSDs underwent PAB with an adjustable FloWatch-PAB^®^.

Criteria for inclusion in this prospective study were infants with the presence of multiple muscular apical VSDs (Swiss cheese type), with or without associated congenital heart defects, including the presence of other restrictive or unrestrictive VSD, either of peri-membranous or inlet type. In the study period no one infant with these characteristics underwent primary repair.

All patients presented with multiple muscular apical VSDs, associated with an additional large mid-muscular VSD in three patients, a large peri-membranous VSD in two and a large inlet VSD in one patient.

The diagnosis was established by echocardiography in all infants, and 9/17 (53%) patients also underwent cardiac catheterization and angiography.

The other associated cardiac anomalies are listed in Table 1.

**Table 1:** **Associated cardiac anomalies**.

Number (%)	Associated cardiac anomaly
12 (71)	Patent ductus arteriosus
2 (12)	Aortic coarctation
2 (12)	Hypoplastic aortic arch
2 (12)	Persistent left superior vena cava
1 (6)	Left isomerism with polysplenia
1 (6)	Cor triatriatum
1 (6)	Partial anomalous pulmonary venous connection

Two infants had undergone previous cardiac surgery, one for closure of patent ductus arterious and the other for closure of patent ductus arterious with aortic coarctectomy, while one infant had undergone interventional closure of patent ductus arteriosus in cardiac catheterization laboratory.

One infant with Down syndrome had previously undergone non-cardiac surgery (colostomy) for an associated gastro-intestinal malformation.

Five infants (5/17 = 29.4%) required pre-operative mechanical ventilation, with a mean duration of 64 days (range 7–240 days), mostly due to the late referral for surgery from other institutions or to the presence of recurrent respiratory infections.

The structural characteristics of the FloWatch-PAB^®^ device, mechanism of functioning, surgical technique of implantation, and the procedures for post-operative percutaneous adjustments, have all been previously reported (32, 33, 36, 37).

At the moment of the beginning of this prospective study the device had already obtained the C.E. mark (equivalent in Europe to the FDA approval for USA), after a multicenter prospective study conducted through hospitals in different European countries (33). Furthermore the FloWatch-PAB^®^ had been successfully implanted by the same surgical team in other congenital heart defects, and therefore there was no need for approval by the institutional ethical committee.

As for any other surgical procedure in the pediatric cardiac service, written informed consent was obtained from the parents or the legal guardian of all patients.

In all the patients described in this study the device was implanted through a median sternotomy.

Nine patients underwent closure of an associated patent ductus arteriosus at time of PAB.

Cardiopulmonary bypass was required in 3/17 (18%) infants, in two for aortic arch reconstruction, and one for repair of cor triatriatum and closure of a large associated peri-membranous VSD.

In order to analyze the results of the study, the following data were prospectively collected with a database prepared before the beginning of the study and continuously updated:

(a) survival
(b) duration of post-operative mechanical ventilation
(c) length of stay in Intensive Care Unit (ICU)
(d) length of stay in hospital
(e) need for re-operation
(f) need for percutaneous adjustments of FloWatch-PAB^®^
(g) interval from implant to removal of FloWatch-PAB^®^
(h) evolution of the multiple muscular VSDs

For all patients the follow-up was censored at the last clinical observation before December 2012.

## Results

There were no early or late deaths during a mean follow-up of 48 months, with a range from 7 to 98 months.

The mean duration of post-operative mechanical ventilation was 2.4 days, with a range from 0 to 6 days.

The mean ICU and hospital stay were 5.6 (range 1–30 days) and 16.8 days (range 7–58 days) respectively.

The relatively long interval (average 3.2 days) between the end of the mechanical ventilation and the discharge from ICU was due to the fact that in both institutions an intermediate step unit between ICU and regular ward (High Dependency Unit) was not available.

The only re-operation required after FloWatch-PAB^®^ implantation was drainage of a pericardial effusion through a sub-xiphoid approach in an infant who developed a pericardial effusion on the eighth post-operative day after device implantation without associated procedure.

All patients required percutaneous adjustments of the device FloWatch-PAB^®^, with a mean of 4.8 times/patient (range from 2 to 9 times) to tighten the banding, and a mean of 1.1 times/patient (range from 0 to 3 times) to lose the banding with the patient’s growth. The decision to adjust the banding was in the immediate post-operative period generally taken with progressive increase of the tightening until reaching the wanted pressure gradient, measured with Doppler echocardiography, providing normal distal pulmonary artery pressure with oxygen saturation>90% on room air at pulse oximeter. In the late follow-up, with interval evaluations dictated by the clinical conditions, adjustments of the banding were required to lose the banding because of the patient growth, with or without concomitant size reduction of the multiple muscular VSDs.

After a mean interval of 29 months, with a range 8–69 months, 10 patients (10/17 = 59%) underwent re-operation for FloWatch-PAB^®^ removal:

(a) seven patients underwent associated procedures: closure of a remaining VSD in six: peri-membranous in three patients, mid-muscular in two, and inlet in one Damus–Kaye–Stansel, bi-directional Glenn, and atrial septectomy in one
(b) three patients required only FloWatch-PAB^®^ removal without need for cardio-pulmonary bypass. In all these 10 patients pre-operative echocardiography and/or cardiac catheterization with angiography demonstrated closure of all the muscular multiple VSDs.

Pulmonary artery reconstruction was not required in 9/10 (90%) patients, even with an interval between FloWatch-PAB^®^ implantation and removal up to 98 months. In only one patient at the time of the FloWatch-PAB^®^ removal the surgeon decided to perform a patch enlargement of the main pulmonary artery because of a residual velocity of 3.2 m/s through the main pulmonary artery on intra-operative echocardiographic Doppler.

In five (5/7) of the remaining patients with FloWatch-PAB^®^ still *in situ*, at the last clinical observation with echocardiography all the muscular VSDs had spontaneously closed. There are residual muscular VSDs, however significantly reduced in size, still present in the two patients both of whom have the shortest follow-up, of 7 and 25 months respectively.

Overall, including the patients with the FloWatch-PAB^®^ still *in situ*, the multiple muscular VSDs had closed in 15/17 (88.2%).

All devices were functioning at the moment of removal, proven by the complete release of the banding before the surgical procedure, as well as at the last follow-up, with device testing under control by Doppler echocardiography.

## Discussion

### Repair

The management of infants with multiple muscular VSDs is complicated by the significant early and late mortality and morbidity when primary surgical repair is undertaken (1, 2, 4–21).

The surgical closure of multiple muscular VSDs remains a technically difficult procedure, due to inadequate exposure of the defects, with or without a right or left ventriculotomy.

The surgical approach was simplified by the anatomical study by Van Praagh showing the communication of the left ventricular apex with the right ventricular apical infundibular recess (11). Since then complete repair has been accomplished by several groups through an appropriately placed right infundibulotomy, providing adequate exposure to the multiple apical muscular VSDs (1, 2, 7–9, 12, 17).

But even a limited right ventriculotomy may cause significant damage to the ventricular muscle with deleterious consequences on cardiac function and rhythm, with subsequent late morbidity and mortality (2, 17).

The surgical approach through a left ventriculotomy continues to have an elevated early mortality (5, 6, 21) and late morbidity and mortality (5).

In addition, the cardiopulmonary bypass required for complete repair in small infants, is still performed with the use of moderate or severe hypothermia in most units. Hypothermia has deleterious effects on the reactive pulmonary vascular resistance, and these effects in association with the presence of a right or left ventriculotomy can make the immediate post-operative course more difficult.

### Hybrid techniques

The poor results initially reported with intra-operative device closure (22–25), with high mortality (14–25%) and failure rates (20–40%) have recently become more encouraging in hospitals with advanced facilities for hybrid techniques (26–30). This alternative technique remains still an option under evaluation in selected institutions.

### Conventional palliation

The palliative technique of conventional PAB not only is complicated by high mortality and morbidity (1, 2, 4–10, 15, 16, 18–21, 31), but in addition is limited by a series of drawbacks, particularly:

(a) difficulty in determining the optimal tightness of the band in the presence of several other peri-operative variables related to general anesthesia with positive pressure ventilation and chest opening
(b) flow adjustment in children with increased pulmonary blood flow, where the PAB is successful in controlling the distal pressure but overflow persists
(c) re-operations frequently required to adjust the band, including some children who quickly outgrow a conventional band
(d) long periods in an intensive care setting with respiratory and/or pharmacological interventions to control pulmonary blood flow
(e) frequent need for a reconstruction of the pulmonary artery at the time of the conventional surgical de-banding for surgical.

To overcome the limitations of conventional PAB, after prolonged experimental studies (32) we introduced the telemetrically adjustable FloWatch-PAB^®^ in 2003. Since then there have been reports also from other institutions as well, with favorable clinical results in different types of congenital heart defects, including palliation for bi-ventricular or uni-ventricular type of repair as well as for left ventricular retraining (33–40).

### Palliation with FloWatch-PAB^®^

The proven advantages provided by the telemetrically adjustable FloWatch-PAB^®^ over conventional PAB are smoother post-operative period, without any requirement for delayed sternal closure, shorter duration of post-operative mechanical ventilation, ICU, and hospital stay (33–35, 37–39).

The improved outcomes with FloWatch-PAB^®^ are the consequence of the telemetrically controlled device, allowing repeated graduated tightening of the PAB, thereby facilitating a controlled and step-wise reduction of the pulmonary blood flow and pressures, tailored to the clinical condition of the patient. In anesthetized infants it is difficult to instantaneously achieve adequate control of pulmonary blood flow and pressure with the application of a fixed conventional PAB: re-adjustments of the band are often required, either with a re-operation or by leaving the chest open for surgical adjustments in the ICU, with substantial risk of mortality and morbidity. This is even more pronounced in very sick infants requiring pre-operative mechanical ventilation and/or additional lesions. The FloWatch-PAB^®^ not only eliminates the need of re-operation to adjust the band but also provides the ability to have precise and progressive tightening of the band over days or weeks when the patient is awake and spontaneously breathing. This can be performed on the ward or outpatient clinic, adjusting the tightening according to the clinical condition and to the trans-band pressure gradient measured with Doppler echocardiography. These are the main reasons why FloWatch-PAB^®^ provides better clinical results over conventional PAB with shorter ICU and hospital stay, fewer complications, and costs reduction (33–35, 37–39).

An additional advantage of the non-circular shape of the cross section of the FloWatch-PAB^®^ is that reconstruction of the pulmonary artery after de-banding is generally not required because the arterial wall remains pliable (36), while following conventional PAB localized fibrosis of the pulmonary artery wall almost always requires an extensive resection with patch reconstruction at the time of de-banding and repair.

Taking into account all these advantages, we decided to evaluate a new treatment strategy of infants with multiple VSDs using the FloWatch-PAB^®^ aimed at minimizing early and late mortality and morbidity.

The results of our multicenter prospective study have demonstrated that this approach provides excellent survival (100%), a smooth post-operative course with short duration of mechanical ventilation, ICU, and hospital stay, with very minimal morbidity.

As the band can be released as the patient grows, the mean interval between first and second stage was 29 months, with a range 8–69 months. This interval not only allows a patient to reach an age and body weight to better tolerate complete repair with lower risk than in the first few weeks or months of life, but showed that in the vast majority (88.2%) of the patients the multiple muscular VSDs had “spontaneously” closed over time, and no surgical closure was necessary.

The reason for the “spontaneous” closure of the multiple muscular VSDs over time has to be found in the hypertrophic reaction of the inter-ventricular septum and the fibrosis accompanying the long duration of the banding *in situ*. This occurred in both groups of patients, with or without associated unrestrictive VSD, either peri-membranous, mid-muscular, or inlet type. Of course, while the first group, without associated unrestrictive VSD, didn’t require any further surgery other than FloWatch-PAB^®^ removal, the patient with associated unrestrictive VSD required subsequent operation to close the residual VSD, but at a later age and higher body weight.

Finally, reconstruction of the main pulmonary artery at the time of FloWatch-PAB^®^ removal was required only in one patient. As demonstrated in all previous reports, the pulmonary artery wall remains pliable even after a long interval period (up to 69 months), allowing total re-expansion and avoiding patch reconstruction (33, 36, 37, 39, 40).

### Limits of the study

This study is mainly limited by the absence of a control group.

During the same period of observation there was no homogeneous group of infants with the same characteristics who underwent surgical repair or conventional PAB.

Because of our previous experience, we decided to offer initial palliation with the FloWatch-PAB^®^ to all infants with this diagnosis, and therefore our comparison has been made with the data provided by the literature.

## Conclusion

This reproducible new strategy with adjustable FloWatch-PAB^®^ simplifies the management of infants with multiple muscular VSDs and provides the following advantages:

(a) good results (0% mortality)
(b) delayed surgery with a high incidence (88%) of spontaneous closure of multiple muscular VSDs
(c) facilitated closure of residual unrestrictive VSD (peri-membranous, mid-muscular, or inlet) at an older age and higher body weight
(d) PAB application and late removal can potentially be the only procedures required for Swiss cheese multiple VSDs without associated peri-membranous unrestrictive VSD.

## Conflict of Interest Statement

The authors declare that the research was conducted in the absence of any commercial or financial relationships that could be construed as a potential conflict of interest.

## Footnotes

^1^www.eactscongenitaldb.org
